# A cross sectional analytic study of modes of delivery and caesarean section rates in a private health insured South African population

**DOI:** 10.1371/journal.pone.0219020

**Published:** 2019-06-27

**Authors:** Geetesh Solanki, Susan Fawcus, Emmanuelle Daviaud

**Affiliations:** 1 Health Systems Research Unit, South African Medical Research Council, Cape Town, South Africa; 2 Honorary Research Associate: Health Economic Unit, University of Cape Town, Cape Town, South Africa; 3 NMG Consultants and Actuaries, Cape Town, South Africa; 4 Department of Obstetrics and Gynaecology, University of Cape Town, Cape Town, South Africa; Medical Research Council, SOUTH AFRICA

## Abstract

**Background:**

Monitoring Caesarean Section (CS) rates is essential to ensure optimal use of the procedure. Information on CS rates in the South African private sector is limited and information from this study will assist in planning for the proposed NHI in South Africa.

**Objectives:**

The objectives of this paper are to assess mode of delivery patterns and to determine CS rates amongst South African private health insurance scheme members; and to assess the extent to which CS rates are influenced by age and health status of the mother.

**Methods:**

The 2015 claims for members of 10 health insurance schemes were analysed to assess delivery type patterns. Mode of delivery patterns were assessed by 6 delivery types: emergency, elective and “other” for caesarean deliveries; and non-assisted, assisted and “other” for vaginal deliveries; as well as by age and health condition of the mother.

**Results:**

Of a total of 6,542 births analysed, 4,815 were CS giving a CS rate of 73·6% (95% CI 72·5%;74·7%). Emergency CS were the most common mode of delivery (39·7%), followed by elective CS (39·5%). CS rates increased with increasing maternal age and were higher for women with a medical condition.

**Conclusions:**

CS rates for the South African private sector are considerably higher than the safe rates recommended by the WHO. The high CS rates is a cause for concern for the health system under the proposed NHI. To support initiatives encouraging evidence based practice, further research is required to understand the drivers for the high CS rates.

## Introduction

Caesarean section (CS) is an important intervention to save the lives of mothers and newborns when performed for specific medical and obstetric indications. However, as for all surgeries it is associated with adverse complications in the short term and for the subsequent pregnancy. Higher than recommended rates of CS can increase risks of negative outcomes as well as being financially inefficient for health systems.

Evidence suggests that most healthy women prefer to give birth via a normal vaginal delivery (NVD) [1], provided safety for them and their baby can be ensured. This option is associated with fewer complications and is more sustainable for health-care systems [2]. The 2015 WHO statement, based on country data, demonstrates that caesarean section (CS) rates above 10–15% conferred no further benefit in reducing maternal and perinatal mortality [3]. However, CS rates are rising worldwide raising debates as to what levels of CS rates are appropriate and the additional short-term and long-term risks and costs associated with inappropriately high CS rates [4] [5]. There are inequities in CS rates between and within countries [6]. For the least developed countries, there is still a need to improve access to safe CS to reduce maternal and perinatal mortality. However, for many other countries CS rates are consistently higher than what is considered medically justifiable [7]. The WHO statement thus further recommends that “Every effort should be made to provide CS to women in need, rather than striving to achieve a specific rate” and based on the finding that maternal mortality is raised threefold for CS compared to vaginal delivery [8], recommends against CS on maternal request. To assist the efforts to reduce, when relevant, the CS rates, the WHO has produced recommendations on non-clinical interventions to reduce unnecessary caesarean sections [9].

Monitoring both CS rates and outcomes is essential to ensure that policies, practices and actions for the optimization of the utilization of CS lead to improved maternal and infant outcomes [4] [3]. CS rates in the South African public sector of 26·2% over the period 2015–2016 [10] would appear to be broadly in line with the rates considered to be justifiable by WHO [11]. In line with global trends [7], CS rates in the South African private sector are generally believed to be much higher. However, this is based on limited evidence. A search of published literature revealed only one publication by Naidoo and Moodley, who reported a CS rate of 65% in 2009 based on an audit of private practice [12]. In addition, a chapter on maternal deaths in the private sector in the 2011–2013 Saving Mothers report gave a CS rate of 67% for the private sectors [13]. To the extent that much of the published literature on CS rates in the private sector is either dated, based on a relatively small sample, or does not systematically examine the underlying factors driving caesarean rates, it is difficult to draw conclusions regarding current CS rates and patterns in the South African private sector. Whereas there is much data on CS rates and indications in the South African public sector, there is less accurate information on the pattern of obstetric service delivery, including CS, in the private sector. Understanding the CS rates in the private sector in South-Africa is of importance in the context of the proposed implementation of the National Health Insurance in South Africa [14]. In terms of the proposals for the NHI, both public and private providers will be contracted to deliver services and the information from this study would be valuable to assess how improved health service coverage can be achieved without unnecessary interventions.

The objectives of this paper are to determine mode of delivery and caesarean rate patterns amongst South African private health insurance scheme members, and to assess the extent to which caesarean rates were influenced by age and health status of the mother.

## Methods

### Study design

The study is a cross sectional analytic study and is based on the analysis of the demographic and claims records of a large sample of South Africans with private health insurance cover.

### Data and data source

For the study, secondary de-identified data were extracted from the medical claims and member records held in the data warehouse of NMG Consultants and Actuaries (NMG), an independent consulting firm providing consulting and actuarial services to South African private health insurance funds covering about half a million people (~5% of the privately insured population). Access to use of the data for the study was obtained via a confidentiality agreement between NMG and the principal investigator. All the data was provided in an anonymized format and the research team had no access to information that would enable the identification of individuals at any time. The data used for this study are not proprietary and will be made available by NMG Consultants and Actuaries to individuals with credible academic/research credentials who want to access the data for scientific and/or academic research purposes and are willing to commit to handling the data in a manner which is consistent with confidentiality requirements.

The average contribution, average health care expenditure and age distribution of the study sample were compared to that of the broader South African population with private health insurance cover. The profiles were found to be broadly comparable ensuring that the findings of the study are generalisable and can be extrapolated to the broader South African population with private health insurance cover (S1 Table).

For this study, all claims submitted for reimbursement of health care services rendered or items dispensed to members of 10 health insurance funds over the period January-December 2015 and from all nine South African provinces were collated and from this collated data claims related to “maternity events”, the unit of analysis for this study, were extracted and analysed. Data for 2015 was used as that was the most recently available data when the data for this study was first collated and prepared for analysis. The population of South Africans with health insurance is stable and there is no obvious reason to suggest that there have been major changes in the patterns of utilisation from 2015 to date.

The data contained the following information for each claim submitted: a unique study individual identifier; the dates for the commencement and completion of the service; a code and description for each service rendered/item dispensed, an ICD-10 (10th revision of the International Statistical Classification of Diseases and Related Health Problems) code for the diagnosis of the condition being treated; a Current Procedural Terminology (CPT) code for the procedure carried out; a National Pharmaceutical Product Index (NAPPI) code for any surgical, medical or consumable item dispensed; and the amount being claimed.

### Data classification

**Fig 1:**
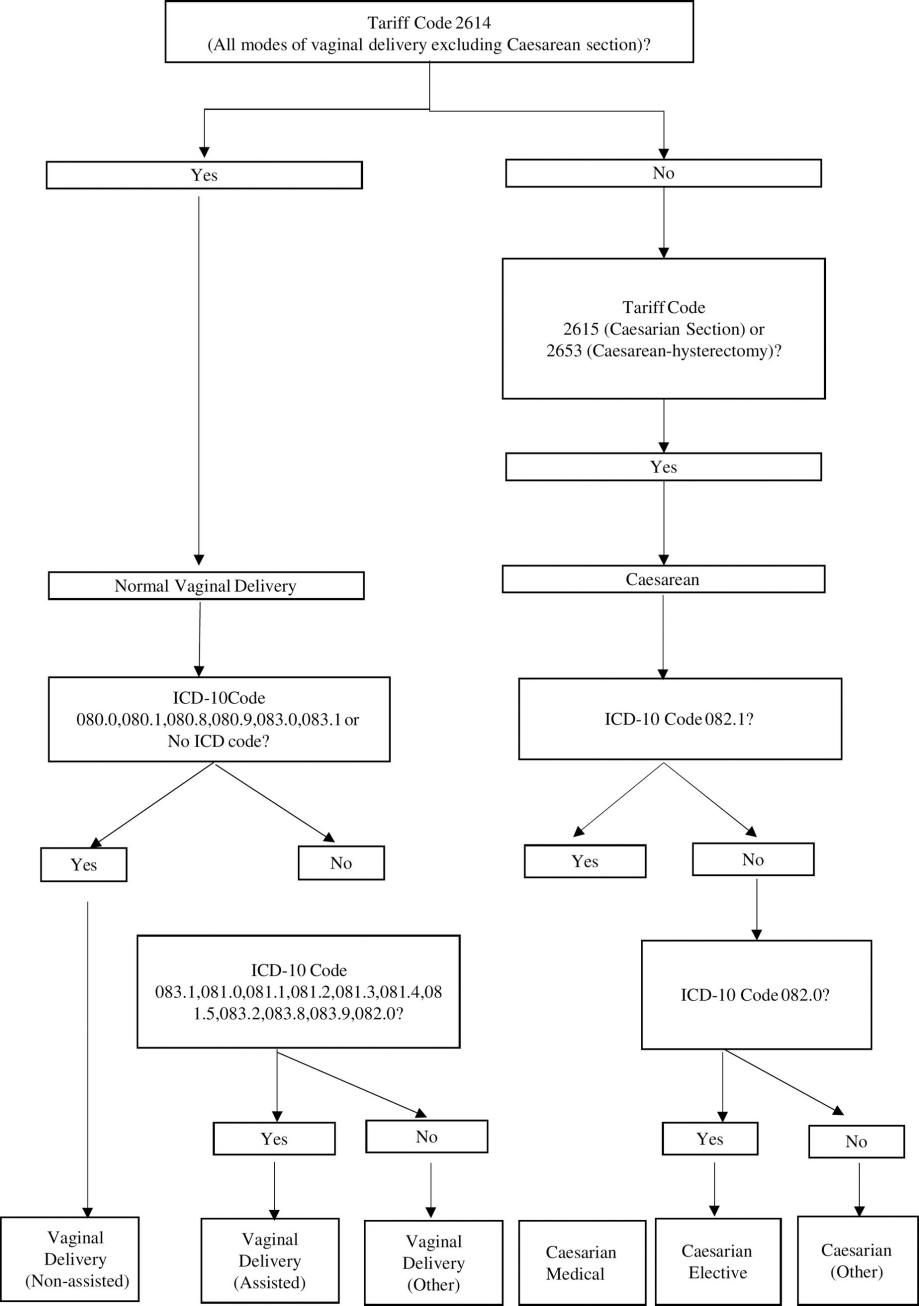
Logic to Identify and classify delivery types.

A two-staged approach using a combination of Tariff Codes and ICD-10 codes was used to identify and classify the maternity events as per the approach summarised in Fig 1. For the first stage, tariff codes were used to identify and classify the maternity events into vaginal and caesarean birth types. For the second stage, ICD-10 codes were used to further categorise births by mode of delivery: non-assisted, assisted and “other” for vaginal births and emergency, elective and “other” for caesarean births. Assisted deliveries are vaginal deliveries assisted by ventouse or forceps.

Due to limitations of the available data, the analysis of delivery types was limited to age and health status. For the health status variable, the “medical condition” category consists of women with one or more of the following: hypertension, diabetes, HIV positive, and the “healthy” category consists of women who did not have any of the conditions. The medical condition variable was created based on the South African Council for Medical Scheme Guideline algorithms for identifying members with medical conditions using claims records [15]. The data set was unable to provide information on the stated indication for CS and/or associated obstetric complications. The average amount claimed by hospitals, gynaecologists, anaesthetists, general medical practitioners and “other” health care providers was determined to assess the relative costs of vaginal and caesarean deliveries.

### Statistical analysis

The proportion of births by mode of delivery and caesarean rates were determined for the entire group and for the following analytic groups: Age (15–19 years, 20–24 years, 25–29 years, 30–34 years, 35–39 years, 40–44 years, 45+years), maternal health status.

The proportion of births by mode of delivery are reported. For each analytic group, the caesarean rate (percentage with 95% confidence intervals (CI)) are reported. For the study, the CS rates are reported as the proportion of total births that were delivered by CS. A logistic regression model was used to test the fully adjusted statistical significance of the association between the exploratory variables (age and health status) and the dependent variable (birth by caesarean section). Odds ratios with 95% confidence intervals (CI) are reported, and a 95% CI excluding 1 was considered statistically significant with the reference groups being 30–34 year age group for age and the healthy group for health status. SAS [16] was used to analyse the data.

For the cost assessment, the average fees claimed with 95% CI per birth overall and by provider type for vaginal and caesarean deliveries are reported.

### Ethical considerations

This research was undertaken by the Health Systems Research Unit of the Medical Research Council of South Africa and data for the study were made available as part of NMG’s commitment to support research initiatives with broader public health significance. The company does not advise its health insurance clients on the clinical treatment of its members. The data were accessed in terms of and under the conditions set out in the consulting agreement between NMG and their client schemes and a confidentiality agreement between NMG and the principal investigator for this study. The data were analysed by NMG internally and were not made available to any other third party. Only secondary, de-identified data was used for the study, all findings are presented at an aggregate level for all the health insurances combined and at no point is confidential scheme or member information disclosed. Ethics approval for the use of the database to carry out this study was granted by the Ethics Committee of the South African Medical Research Council project registration number EC001-1/2019.

## Results

### Sample description

The total dataset comprised the claims of a total of 519,458 individuals (279,648 women) over the period January to December 2015. From this dataset, the claims data related to a total 6,542 maternity events were extracted and analysed for this study. Of the total 6,542 births, 87·6% were to women aged between 25 and 39 years with 7·4% to women under the age of 25 years and 5% to women over the age of 39 years. Births to women with medical conditions made up 4·2% of the births.

### Mode of delivery patterns

The distribution of births by mode of delivery is presented in Table 1. The overall caesarean section rate was 73.6%. Of the 4815 CS births, 1912 (39.7%) were Emergency, 1904 (39.5%) were Elective, and for 999 (20.7%) it was “other” (unknown or unspecified). Of the 1727 (26·4%) vaginal births, 1589 (92.0%) were unassisted, 55 (3.2%) were assisted; and 83 (4.8%) were “other” (unknown or unspecified). Of the 55 assisted vaginal births, 54 were vacuum deliveries and 1 was a forceps delivery.

**Table 1:** Births by mode of delivery.

Delivery Mode		N	% Caesarean/ Vaginal	% All Deliveries	Epidural use	
**Caesarean Deliveries**	Emergency	1912	39.7%	29.2%	81	4.2%
	Elective	1904	39.5%	29.1%	54	2.8%
	Unknown/Unspecified	999	20.7%	15.3%	23	2.3%
** **	**Total Caesarean**	**4815**	**100.0%**	**73.6%**	**158**	**3.3%**
**Vaginal Deliveries**	Unassisted	1589	92.0%	24.3%	907	57.1%
	Assisted	55	3.2%	0.8%	32	58.2%
	Unknown/Unspecified	83	4.8%	1.3%	29	34.9%
** **	**Total Vaginal**	**1727**	**100.0%**	**26.4%**	**968**	**56.1%**
**Total**		**6542**		**100.0%**	**1126**	**17.2%**

For the group was a whole, Emergency CS was the most common mode of delivery (1912, 29.2%), followed by elective CS (1904, 29.1%), vaginal delivery unassisted (1589, 24.3%), caesarean unknown (999, 15.3%), vaginal delivery other (83, 1.3%) and vaginal delivery assisted (55, 3.2%).

Epidural anaesthesia was provided for 968 (56·1%) of women having vaginal delivery. Considered by type of vaginal delivery, the rates were 57·1%, 58.2% and 34·9% for vaginal unassisted, vaginal assisted and other respectively.

### Caesarean rates by age and health status

Caesarean rates by age group and health status are presented in Table 2. The overall CS rate of 73·6%(95% CI: 72·5%; 74·7%) was notable. CS rates increased with increasing age, with the 63·0% (95% CI: 58·7%; 67·3%) rate for women less than 25 years of age being the lowest and the 80·6% (95% CI: 76·3%; 84·9%) rate for 40+ year old women being the highest. Compared to the CS rates of women aged 30–34 years, the rates for women under 25 years of age (OR = 0·6, 95% CI: 0·5; 0·7) and women aged 25–29 years (OR = 0·7, 95% CI: 0·6; 0·8) were significantly lower. The 90·2% CS rates for women with a medical condition was significantly higher than the 72·9% rate for women without medical conditions (OR = 3·1, 95% CI: 2·1; 4·7). Women aged 35 to 39 years had the highest proportion of elective caesarean birth. Women with a medical condition also had a higher proportion of elective caesarean births (OR = 1·3, 95% CI: 1·0; 1.7).

**Table 2:** All caesarean and elective rates by age and health status.

	Total Births		All Caesarean Births			Elective Caesarean Births		
	N	% of Total	N	Rate (95% CI)	OR vs Reference Group (95% CI)	N	Rate (95% CI)	OR vs Reference Group (95% CI)
**By Age Group:**								
<25 years	484	7.4%	305	63.0% (58.7%-67.3%)	0.6 (0.5–0.7)	91	18.8% (16.5%-21.2%)	0.5 (0.4–0.6)
25–29 Yrs	1816	27.8%	1252	68.9% (66.8%-71.1%)	0.7 (0.6–0.8)	415	22.9% (21.6%-24.1%)	0.6 (0.6–0.7)
30–34 Yrs*	2627	40.2%	1992	75.8% (74.2%-77.5%)	**1.0**	836	31.8% (30.8%-32.9%)	**1.0**
35–39 Yrs	**1290**	**19.7%**	**1004**	**77.8% (75.6%-80.1%)**	1.1 (0.9–1.3)	458	35.5% (34.0%-37.0%)	1.2 (1.0–1.4)
40+ Yrs	325	5.0%	262	80.6% (76.3%-84.9%)	1.2 (0.9–1.6)	104	32.0% (29.3%-34.7%)	1.0 (0.8–1.3)
**By Health Status:**								
Healthy*	**6267**	**95.8%**	**4567**	**72.9% (71.8%-74.0%)**	**1.0**	**1810**	**28.9% (28.2%-29.6%)**	**1.0**
Medical Condition	275	4.2%	248	90.2% (86.7%-93.7%)	3.1 (2.1–4.7)	94	34.2% (32.0%-36.3%)	1.3 (1.0–1.7)
**Total**	**6,542**	**100%**	**4815**	73.6% (72.5%-74.7%)		**1860**	**28.4% (27.8%-29.1%)**	

* Reference Group

### Fees for vaginal and caesarean deliveries

The average fees by provider type for vaginal and caesarean deliveries is presented in Table 3. The average overall cost of ZAR 38,918 caesarean deliveries was significantly higher than the average cost of ZAR25,662 for vaginal deliveries. Of the total difference of ZAR13,256 in the cost per case between caesarean and vaginal deliveries, differences in hospital costs (ZAR8,428) and gynaecologist’s costs (ZAR1,950) were the largest contributors. Although not presented in the table we also examined the overall average fees by mode of delivery and whether epidural anaesthesia was applied or not. There was little difference in the average cost per case between unassisted and assisted vaginal deliveries with or without epidural anaesthesia. The average cost for unassisted vaginal deliveries was ZAR25,488 with epidural anaesthesia and ZAR25,312 without epidural anaesthesia. The average cost for assisted vaginal deliveries was ZAR25,574 with epidural anaesthesia and ZAR25,357 without epidural anaesthesia.

**Table 3:** Average fees for vaginal and caesarean deliveries by provider type.

Provider Type	Average Fees (ZAR) Per Case (95% CI)	
	Vaginal Delivery	Caesarean
Hospital Fees	18311 (17893–18730)	26739 (25894–27584)
Gynaecologist	5336 (5166–5505)	7285 (7133–7437)
Anaesthetist	1051 (931–1172)	2074 (2007–2140)
GP	304 (247–362)	1524 (1456–1591)
Other	659 (508–811)	1296 (1213–1379)
**Total**	**25662 (25056–26267)**	**38918 (38033–39803)**

## Discussion

Using a large representative sample, the study confirms that CS rates in the South African private sector is high and considerably higher than the rates reported in other countries [4, 7] and the rates considered to be justifiable by WHO [3]. The CS rate in South African private sector is also three times higher than that reported in South African public sector [10]. This high rate is a cause of concern given the potential for short and long-term complications, and the impact on health system resources. In terms of identifying an ‘ideal CS rate’ for a population, it is important to go beyond WHO’s recommendations based only on mortality reduction. Consideration of what rates are justifiable should also consider the reduction of maternal morbidity (such as third-degree tears, obstructed labour, post-partum sepsis) and perinatal morbidity (such as neonatal encephalopathy, neonatal jaundice) [17]. This means that a CS rate above 10–15% may be justifiable to prevent the maternal and perinatal morbidities described above. The focus of the WHO statement is that women should have access to safe CS according to need. The 26% CS rate in the South African public sector maybe appropriate even though higher than the 10–15%, if morbidity is considered. However, it is difficult to justify the 73·6% CS rate in the private sector even if morbidity prevention is considered as the burden of obstetric and perinatal pathology in the private sector is likely to be less than in the public sector, given the higher HIV prevalence and reduced access to care of women in the public sector. The SA private CS rates are also more than those in well-functioning national health systems with highly skilled specialists as in the UK and Netherlands and which have similar or better outcomes than the SA private sector in terms of maternal and perinatal mortality [18].

The high CS rate in the South African private sector and the fact that a large proportion of them are being carried out as elective procedures amongst young women with no medical condition, rather than as emergencies is a cause for concern. It raises the question as to what the obstetric indications for CS were, whether they were evidence based, or whether they were done on maternal request only. The finding that the CS rate was higher in women with medical conditions such as hypertension and diabetes is expected because these conditions may require CS. What is surprising is the low overall rate of medical problems in the population (4·2%), given the very high CS rate of 73·6%. Medical conditions are more common in older women which would partially explain the association between advanced age and CS rate.

Although not the purpose of the study, it is interesting to note the low proportion of assisted vaginal deliveries; 3.2% of vaginal deliveries, and 0·8% of total deliveries. Assisted vaginal deliveries are those done by ventouse or forceps and in this case series were mostly ventouse. This overall assisted delivery rate of 0.8% is slightly lower than the 1% in the public sector of South Africa [19]; and much lower than in Europe or USA where it is in the range of 10–15% [20, 21]. This is despite the high rate of epidural anaesthesia use (56.1%) in the SA private sector in labouring women. Epidural anaesthesia is known to be associated with greater need for assisted delivery in the second stage [22]. The reasons for this surprisingly low assisted delivery rate cannot be ascertained from this study but may reflect earlier recourse to first or second stage CS, lack of skills and medico legal considerations.

Globally, economic pressure on hospitals, financial incentives, private health insurance, and fear of lawsuits, in the context of rising medico-legal claims have been identified as drivers of high and increasing CS rates [16]. Most private obstetrics practitioners in South Africa operate on a solo basis. This contrasts with the public sector where maternity care is organised around a maternity team made up of specialists, junior doctors and midwives. Within this context, delivering via CS rather NVD would be the more attractive option for private practitioners for a number of reasons. CS can be scheduled at a time that is convenient to the practitioner and the delivery time is more predictable [23]. This means they can conduct their other responsibilities such as outpatient consultations or gynaecological surgery without risk of interruption. Time considerations are particularly important in the South African private sector context given that the professional fees are not time -based. The professional fees for delivery by CS is 37% higher than that for NVD’s–for this study the average professional fee was R7285 per CS delivery compared to a fee of R5336 per NVD. With high and rapidly rising medical malpractice indemnity fees for obstetricians in South Africa [24] delivery by CS is perceived as a lower risk option as legal claims for hypoxic brain injury are very high. Demand for CS from expectant mothers, due to fear of labour exacerbated by stories of negative birth experiences could also play a role [25, 26]. While evidence suggests that Midwife led obstetrics is associated with less intervention and lower CS rates [27], private obstetrics in South Africa is doctor dominated with a very small proportion of births being midwife led. Although there are some private midwifery practices, they are finding it increasingly difficult to find private obstetricians willing to work with them for referrals in instances of emergencies However, the nature of the data used for this study cannot confirm which of these factors are the key drivers for the high CS rates in the South African private sector.

## Limitations

The data used for the study was for 2015 and although unlikely, delivery patterns may have changed since that time. The data available was limited to types of birth, age and some indicators of health status of the women. Since it was aggregated secondary data, it did not provide details of obstetric complications or specific indications for CS. This meant that the clinical basis for the high CS rates in the private sector could not be elucidated. This is not possible to ascertain from ICD 10 codes, the completion of which is not always adequate. The ICD10 codes do not accurately reflect disease burden or the lack thereof. For 20.7% of CS it was not specified whether the CS was emergency of elective, further limiting interpretation of the data. The data does not include private midwife deliveries.

## Conclusion

The South African Society of Gynaecologists (SASOG) have launched the Better OBs program to encourage evidence-based practice and facility audit processes. The reasons for the high CS rates in the private sector in South Africa and the possible ways in which this can be addressed should be explored and researched as part of this program. The program should also explore the extent to which the recommendations of the International Federation of Gynaecology and Obstetrics (FIGO) to reduce unnecessary CS [23] can be taken forward in the South African private sector context. These recommendations include, review of the fee structure for CS vs NVDs, the monitoring of CS rates by hospitals, use of the uniform Robson/WHO classification system [28], properly informing women on the benefits and risks of CS, and investing the savings from reduction in CS rates to improve intrapartum care. Opportunities for the re-organisation of the private obstetrics to be more team oriented, multidisciplinary and with an increased role for midwifes should also be explored. In developing strategies, the complexity of the factors that drive overuse should be recognised, and approaches of focusing on single interventions that target only one driver should be avoided [29]. There is an urgent need to identify and address drivers for the high CS rates in the South African private sector. The South African government is forging ahead with plans to implement a National Health Insurance (NHI) [14]. As proposed, the NHI will rely on a mix of public and private providers and it is important that the high CS rates in the private sector are not transposed to the public sector. Rather, the CS skills and expertise of private sector should be used to assist the public sector where there is currently a lack of capacity.

## Supporting information

S1 Table Characteristics of study sample vs. broader south african health insured population. (DOCX)
