# Supplementary material for: A cross sectional analytic study of modes of delivery and caesarean section rates in a private health insured South African population
Source: PLoS One. 2019 Jun 27;14(6):e0219020. doi: 10.1371/journal.pone.0219020 (PMC6597103; doi:10.1371/journal.pone.0219020)
Supplement: S1 Table — (DOCX) [file pone.0219020.s001.docx]

**S1 Table: Characteristics of Study Sample vs. Broader South African Health Insured Population**

|  | **Study Population** | **Total**  **Population**** |
| --- | --- | --- |
| Total Lives | 516677 | 8839892 |
| Total Families | 264998 | 3749493 |
| Average Age | 36·0 | 33·0 |
| Average Contributions* | 19101 | 18386 |
| Average Health Care Expenditure* | 18188 | 16239 |
| * ZAR Per life per year | | |
| ** Excluding Study Population | | |
